# Tumor-Derived CXCL10 and CCL20 Polarize the Immune Microenvironment in Nasopharyngeal Carcinoma via Competitive Recruitment of Effector T Cells and Tregs

**DOI:** 10.7150/ijms.116010

**Published:** 2026-01-01

**Authors:** Benjian Zhang, Xiaotian Yuan, Lai Meng, Yunqing Liu, Yaxuan Wang, Zirong Chen, Haoxiang Zeng, Xinyue Zhang, Zhouying Peng, Hua Zhang, Weihong Jiang

**Affiliations:** 1 Department of Otolaryngology Head and Neck Surgery, Xiangya Hospital of Central South University, Changsha, People's Republic of China.; 2 Department of Otolaryngology Head and Neck Surgery, Guangxi Medical University Cancer Hospital, Nanning, People's Republic of China.; 3 Hunan Province Key Laboratory of Otolaryngology Critical Diseases, Xiangya Hospital of Central South University, Changsha, People's Republic of China.; 4 National Clinical Research Center for Geriatric Disorders, Xiangya Hospital of Central South University, Changsha, People's Republic of China.; 5 Anatomy Laboratory of Division of Nose and Cranial Base, Clinical Anatomy Center of Xiangya Hospital, Central South University, Changsha, People's Republic of China.; 6 Department of Oncology, Xiangya Third Hospital of Central South University, Changsha, People's Republic of China.

**Keywords:** nasopharyngeal carcinoma, CXCL10, CCL20, Treg, tumor immune microenvironment

## Abstract

Nasopharyngeal carcinoma (NPC) exhibits a heterogeneous tumor immune microenvironment (TIME) shaped by chemokine signaling, yet the functional roles of tumor-derived chemokines remain elusive. This study integrates single-cell RNA sequencing (scRNA-seq) of NPC tissues with validation in 109 primary patient samples, revealing CXCL10 and CCL20 as tumor-secreted chemokines localized to distinct malignant epithelial subpopulations with antagonistic roles. CXCL10 correlates with prolonged progression-free survival (PFS) and enriches an immune-active TIME by recruiting CD8+ T cells and CD20+ B cells, whereas CCL20 associates with poor prognosis and immunosuppression through preferential recruitment of regulatory T cells (Tregs). Functional validation via in vitro chemotaxis assays and in vivo xenograft models demonstrates that CXCL10 overexpression suppresses tumor growth by enhancing effector immune cell infiltration, while CCL20 promotes Treg accumulation without impeding tumor progression. Mechanistically, Tregs in NPC exhibit elevated expression of co-inhibitory molecules (CTLA4, TIGIT) and engage B cells via CTLA4-CD86 signaling, potentially impairing antigen presentation. Multi-omics analysis of bulk RNA-seq, immunohistochemistry, and cell-cell communication further delineates the antagonistic interplay between CXCL10-driven immune activation and CCL20-mediated immunosuppression. Our findings establish CXCL10 and CCL20 as dual regulators of TIME polarization in NPC, offering prognostic biomarkers and therapeutic targets to rebalance antitumor immunity.

## Introduction

Nasopharyngeal carcinoma (NPC) is a malignant tumor originating from the epithelium of the nasopharyngeal mucosa, with a higher incidence in Southeast Asia and southern China^1^. NPC is strongly associated with Epstein-Barr virus (EBV) infection, genetic factors, and environmental exposures, and it demonstrates distinct geographical and ethnic patterns^2^. Compared with other head and neck cancers, NPC is often diagnosed at an advanced stage due to its subtle early symptoms and has a high risk of lymph node and distant metastasis^3^. Although radiotherapy and chemotherapy are the main treatments, patients with recurrent and metastatic NPC often face poor prognosis due to treatment resistance^4^.

In recent years, the tumor immune microenvironment (TIME) of NPC has become a focal area of research. NPC's TIME is rich in various immune cells, including T cells, B cells, macrophages, and dendritic cells, which are closely associated with tumor development and progression^5^. Specifically, the upregulation of immunosuppressive cells, such as regulatory T cells (Tregs), and immune suppressive factors like PD-L1 often contributes to immune evasion, inhibiting the body's anti-tumor immune responses^6^-^8^. Studies have shown that the spatial distribution and differentiation state of tumor-associated B cells (such as FCRL4^+^ B cells) and CD8^+^ T cells in NPC may impact patient prognosis and the effectiveness of immunotherapy^9^-^11^. Understanding NPC's TIME can offer insights into its immune escape mechanisms and aid in developing more effective immunotherapy strategies.

Chemokines are small protein molecules that, by binding to their respective receptors, induce the migration, localization, and activation of immune cells, playing a critical role in maintaining immune system balance and function^12^, ^13^. In the TIME, the expression of chemokines and their receptors is often dysregulated, resulting in the recruitment and distribution of various immune cells that influence tumor growth and progression^14^. Chemokines can have dual functions within the TIME: they may enhance anti-tumor immune responses by recruiting cytotoxic immune cells, such as effector T cells and natural killer (NK) cells^15^-^17^, or they may facilitate tumor immune escape by attracting immunosuppressive cells like Tregs and myeloid-derived suppressor cells (MDSCs)^18^. For example, CXCL9 and CXCL10 recruit effector T cells into the tumor via CXCR3^19^, ^20^, promoting anti-tumor responses, while CCL2 recruits MDSCs through CCR2, thereby inhibiting immune responses^21^. Tumor cells themselves can also play a critical role in their growth, metastasis, and immune evasion by secreting chemokines. These tumor-secreted chemokines not only directly affect tumor cell proliferation and migration but also attract various immune and stromal cells, creating a TIME that promotes tumor growth and progression^18^, ^22^. For instance, the chemokine CXCL12 (SDF-1), secreted by tumor cells, binds to the CXCR4 receptor to promote invasion and metastasis while recruiting stromal and endothelial cells to support neovascularization^23^. The secretion of chemokines is also closely linked with epithelial-mesenchymal transition (EMT), which enhances tumor cell migratory and invasive potential^24^. Therefore, targeting chemokines and their signaling pathways is a promising strategy for inhibiting tumor progression, reversing immune suppression, and improving the efficacy of immunotherapy.

## Materials and Methods

### Clinical Samples

The human tissue microarrays used and collected in this study were obtained from Outdo Biotech Co., Ltd. (Shanghai, China). Tissue sampling was conducted following the guidelines of the International Organization of Medical Sciences and the World Health Organization and was approved by the ethics committee of Outdo Biotech Co., Ltd. (Shanghai, China) (approval number: HLugA180Su07). All specimens were surgically removed or biopsied for therapeutic purposes, and informed consent was obtained from all patients participating in the study.

### Acquisition and Analysis of Bulk RNA-seq Data

Use R to complete these analyses^25^. The RNA sequencing dataset GSE102349 of NPC was downloaded from the Gene Expression Omnibus (GEO) database, which included 88 primary NPC patients with paired progression-free survival (PFS) information. We also downloaded the transcriptomic data and overall survival information of head and neck squamous cell carcinoma (HNSCC) patients from the The Cancer Genome Atlas (TCGA) database. The raw expression matrix was converted to transcripts per million (TPM) and normalized using the 'limma' package. Mean comparisons and survival analyses were performed using the 'ggplot2', 'ggpubr', 'corrplot', 'survival', and 'survminer' packages.

### Acquisition and Analysis of Two single-cell sequencing (scRNA-seq) Data

Use R to complete these analyses. Two scRNA-seq datasets, GSE150825 and GSE150430, were downloaded from the GEO database. Primary NPC samples, nasopharyngeal lymphatic hyperplasia (NLH), and peripheral blood mononuclear cell (PBMC) samples were extracted from these datasets. Basic single-cell data processing was performed using Seurat V4. Quality control criteria included: 1) Genes expressed in ≥3 cells; 2) Cells with ≥300 and <3000 features; 3) Cells with mitochondrial gene reads accounting for <10% of the transcriptome; 4) Cells with count numbers <10,000. The SCTransform function was used for data normalization and standardization, followed by Principal Component Analysis (PCA) for each cell. DoubletFinder was used to remove doublets based on the top 20 PCs. 3,000 Highly Variable Genes (HVGs) were selected for subsequent analyses. PCA was performed, and batch effects were corrected using the 'harmony' tool. Clustering analysis was conducted using the K-Nearest Neighbors (KNN) algorithm based on manifold learning. Differential gene expression among different cell populations was compared using the Wilcoxon rank-sum test, with significance criteria of adjusted p-value <0.05 and log2FC ≥0.25. Significantly expressed marker genes for each cell population were identified and annotated using the cellmarker online database, along with reference to previous NPC single-cell sequencing studies. Copykat was used to calculate genome copy number variations in individual cells to differentiate between diploid (normal) and aneuploid (tumor) cells. Cytotrace was used to infer cell developmental trajectories, and the slingshot algorithm was employed to predict cell developmental branch structures. Single-sample Gene Set Enrichment Analysis (ssGSEA) with the GSVA package was used to assess gene set enrichment in individual samples, and cellchat was used to infer communication networks between cell subpopulations.

### Immunohistochemistry (IHC)

Freshly excised tissue samples were fixed in 10% formaldehyde for over 24 hours, dehydrated, cleared, embedded in paraffin, and sectioned for pathology slides. Sections were deparaffinized before staining, and antigen retrieval was conducted by heating in Tris-EDTA antigen retrieval solution. Sections were blocked with 20% BSA at room temperature for 30 minutes. Primary antibodies diluted in PBS were applied and incubated overnight at 4°C. After removing the primary antibody solution, PBS-diluted secondary antibodies were added and incubated at room temperature for 50 minutes. HRP-conjugated secondary antibodies were used, followed by DAB substrate application to visualize staining. The staining time was monitored under a microscope, and hematoxylin was used for counterstaining. Stained slides were scanned using a brightfield scanner. Immunohistochemistry scoring criteria included: 1) *Intensity Score*: 0 (negative), 1 (+), 2 (++), 3 (+++); 2) *Proportion Score*: Proportion of cells with an intensity score > 0 in all cells; 3) *Overall Score*: the product of *Intensity Score* and *Proportion Scores*. The slides were independently evaluated by two pathologists, with an inter-rater reliability kappa coefficient exceeding 0.8, and the final results were determined through consensus discussion between the two evaluators. The optimal cutoff value for the Overall Score was determined using Youden's index, with tissue samples subsequently categorized into high-expression and low-expression groups for analysis.

### Immunofluorescence

Freshly excised tissue samples were fixed in 10% formaldehyde for over 24 hours, dehydrated, cleared, embedded in paraffin, and sectioned. Sections were deparaffinized, and antigen retrieval was conducted using Tris-EDTA solution. Blocking was performed with 20% BSA at room temperature for 30 minutes. Antibodies were incubated following the Opal 7-color Manual IHC Kit (NEL801001KT, PerkinElmer) protocol. Tris-EDTA solution was used to remove antibodies between steps, with repeated blocking, incubation, and staining until all target proteins were labeled. Slides were incubated with DAPI for 5 minutes at room temperature, washed with TBST, and mounted with anti-fade medium. Fluorescence scanning was performed. We performed quantitative analysis of multiplex immunofluorescence using Image J software, with the Trainable Weka Segmentation plugin employed for cell segmentation and counting: CD20+ B cells (CD20+CD8-FOXP3-PANCK-), CD8+ T cells (CD20-CD8+FOXP3-PANCK-), and FOXP3+ Tregs (CD20-CD8-FOXP3+PANCK-). For CD20+ B cells and CD8+ T cells, tissues with ≥100 cells/mm³ were considered positive, whereas for FOXP3+ Tregs, a threshold of ≥50 cells/mm³ was used to define positivity.

### Human PBMC Isolation

Whole blood was diluted with PBS buffer at a 1:1 ratio, and ficoll (Cytiva) was slowly added along the tube wall. Samples were centrifuged at 3,000 rpm for 20 minutes at 4°C, and the interface above the ficoll layer was collected. Samples were washed with PBS three times, resuspended in ACK lysing buffer (NCM Biotech), incubated at room temperature for 1 minute, washed again with PBS, and used for subsequent experiments.

### Cell Culture

The human NPC cell line (5-8F) was obtained from the cell bank of the Hunan Key Laboratory of Otorhinolaryngology Major Diseases and verified by short tandem repeat (STR) profiling. Cells were cultured in RPMI 1640 medium containing 10% fetal bovine serum (FBS), penicillin-streptomycin-gentamicin, at 37°C in a 5% CO2 incubator.

### Transwell Assay

PBMCs were resuspended in serum-free RPMI 1640 medium at a concentration of 5×10^6 cells/mL. 200 μL of cell suspension was added to the upper chamber of the transwell, while the lower chamber contained serum-free RPMI 1640 medium with or without 20 ng/ml CXCL10 or 20 ng/ml CCL20. The transwell membrane pore size was 0.4 μm. Cells were cultured for 24 hours, and those in the lower chamber were collected for flow cytometry analysis.

### Flow Cytometry

NPC tissue samples were washed with PBS, minced, and homogenized, then filtered through a 40 μm mesh and treated with ACK lysing buffer to obtain a single-cell suspension. The lower chamber fluid from the transwell assay was also collected and washed with PBS. Cells were blocked with human Fc receptor blocking antibody for 30 minutes, stained with fluorescence-labeled antibodies, fixed with 4% paraformaldehyde, and resuspended in 100 μl of staining buffer for analysis. Data were analyzed using FlowJo software. Antibodies and dyes used included anti-CD4-eFluor450 (Invitrogen), anti-CD8A-eFluor506 (Invitrogen), anti-CD25-PE-eFluor610 (Invitrogen), anti-CD127-Super Bright 702 (Invitrogen), anti-CD20-Pacific Blue (Biolegend), anti-CD19-BV480 (BD Biosciences), and Zombie NIR (Biolegend), anti-CCR6-PE/Dazzle 594 (Biolegend), anti-CXCR3-BV711 (BD Biosciences).

### Plasmid Amplification

The pcDNA3.1-EGFP-Puro lentiviral vector encoding CXCL10 or CCL20 was synthesized by PULLEN Biotech (Guangzhou, China). TOP10 competent E. coli were transformed with the target plasmid, incubated on ice for 20 minutes, heat-shocked at 42°C for 45 seconds, and placed back on ice for 2-3 minutes. The bacteria were resuspended in 500 μl LB medium and recovered at 37°C for 1 hour. 100 μl of bacteria were plated on LB agar plates with ampicillin and cultured at 38°C for 12 hours. A single colony was selected and expanded with ampicillin screening in a 37°C shaker for 12 hours. Bacterial cells were collected by centrifugation at 10,000g for 1 minute, and plasmid extraction was performed using the GoldHi EndoFree Plasmid Midi Kit (CWBIO, Taizhou, China) following the manufacturer's protocol. Plasmids were eluted and stored at -20°C.

### Virus Packaging and Lentiviral Infection

293T cells were expanded to 80% confluency, and the medium was replaced with fresh DMEM. Lipofectamine 3000 (Invitrogen) was mixed with opti-MEM (Gibco) to form solution A. The target plasmid, packaging plasmid, and P3000 were mixed with opti-MEM to form solution B. After a 5-minute incubation, solutions A and B were combined and allowed to rest for 20 minutes, then added to the 293T culture dish. After 8 hours of transfection, the medium was replaced with complete DMEM. After 24 hours of transfection, the viral supernatant was collected and mixed with RPMI 1640 complete medium in a 1:1 ratio. This mixture was added to primary fibroblasts at 40% confluency and allowed to infect for 48 hours. Cells were further cultured with puromycin selection for 1 week to confirm transfection efficacy.

### Western Blot

Treated cells were collected and lysed with RIPA buffer, followed by incubation on a shaker at 4°C for 15 minutes. The protein concentration in the lysates was determined using a Bicinchoninic Acid (BCA) Protein Assay Kit (Beyotime). Proteins were separated using 10% SDS-PAGE and transferred to a PVDF membrane. The membrane was incubated with primary antibodies, followed by incubation with HRP-conjugated goat anti-rabbit secondary antibodies (Proteintech). The immune response bands were detected using Enhanced Chemiluminescence (ECL) reagent (Beyotime). GAPDH was used as an internal control. The primary antibodies used included: anti-CXCL10 (Proteintech), anti-CCL20 (Proteintech), and anti-GAPDH (Proteintech).

### RT-qPCR

Cells were collected, washed twice with cold PBS, and lysed in 1 mL TRIzol for 5 min at room temperature. After adding 200 μL chloroform and centrifugation (12,000 rpm, 15 min, 4°C), the aqueous phase was transferred and mixed with isopropanol to precipitate RNA. The pellet was washed with 75% ethanol, air-dried, and resuspended in RNase-free water. RNA purity (A260/A280: 1.8-2.0) was verified. cDNA was synthesized using the SweScript RT II kit (20 μL reaction: 1 μg RNA, 5× gDNA Remover Mix, 5× RT Mix, RT Enzyme Mix) under the following conditions: 42°C (2 min), 25°C (5 min), 42°C (30 min), and 85°C (5 min). qPCR was performed with Fast SYBR Green Master Mix (20 μL: 2 μL cDNA, 0.4 μL each primer [10 μM], 10 μL master mix, 7 μL water). Cycling conditions: 95°C (30 sec), followed by 40 cycles of 95°C (10 sec) and 60°C (30 sec). Technical triplicates were run, with GAPDH as the reference gene. Relative expression (2-ΔΔCt) was analyzed using GraphPad Prism. The qPCR primer sequences were as follows: IL10 forward (GGGCACCCAGTCTGAGAA) and reverse (GCAACCCAGGTAACCCTTAAAGT); IL21A forward (ACAGCGTGATGTCGAACACT) and reverse (GAGATGAGGTACCATCGCCC); IL34 forward (CTGCCCGTGGCCCTTAG) and reverse (TGAAATCTGGCTCTGTTCACG); TGFβ forward (TGGTGGAAACCCACAACGAA) and reverse (GAGCAACACGGGTTCAGGTA); FASL forward (TACCAGCCAGATGCACACAG) and reverse (GGCATGGACCTTGAGTTGGA); TIGIT forward (TCACACCTACCCTGATGGGAC) and reverse (TGAGGGCTTTCTTCTTTCTAGTCA).

### Animal Experiments

Animal experiments were approved by the Animal Ethics Committee of Central South University (approval number: CSU-2024-0264). Logarithmic phase 5-8F cells were digested, centrifuged, and resuspended in PBS to a final concentration of 2×10^7 cells/ml. 100 μl of cell suspension was injected subcutaneously into the right axilla of 6-week-old NSG mice. On days 7 and 21, human PBMCs (total cell number of 10^7) were administered via tail vein injection in a volume of 200 μl. On day 35, the mice were euthanized, and tumor tissues were harvested, weighed, and either fixed for embedding or prepared into single-cell suspensions.

### Statistical Analyses and Data Visualization

Statistical analyses and data visualization were performed using R and GraphPad Prism 10. The R packages ggplot2 and ggsignif were used for statistical testing and for comparing means or medians. Survival analysis was conducted using the survival package. Patients were stratified into high and low groups based on the median value of gene expression or cell abundance scores, and survival differences between the groups were assessed using the log-rank test. Heatmaps and hierarchical clustering were generated using the pheatmap package and its built-in algorithms. For statistical testing, unpaired t-tests were applied to normally distributed data with equal variances, while the Wilcoxon rank-sum test was used for non-normally distributed data. Paired t-tests were used for paired samples. One-way analysis of variance (ANOVA) was performed for multiple group comparisons, followed by Student-Newman-Keuls (SNK-Q) tests for pairwise comparisons. Statistical significance was defined as p < 0.05 (*), p < 0.01 (**), p < 0.001 (***) and p < 0.0001 (****).

## Results

### Differential Expression of Chemokines in NPC Tumor Cells and its Prognostic Implication

**Figure 1:**
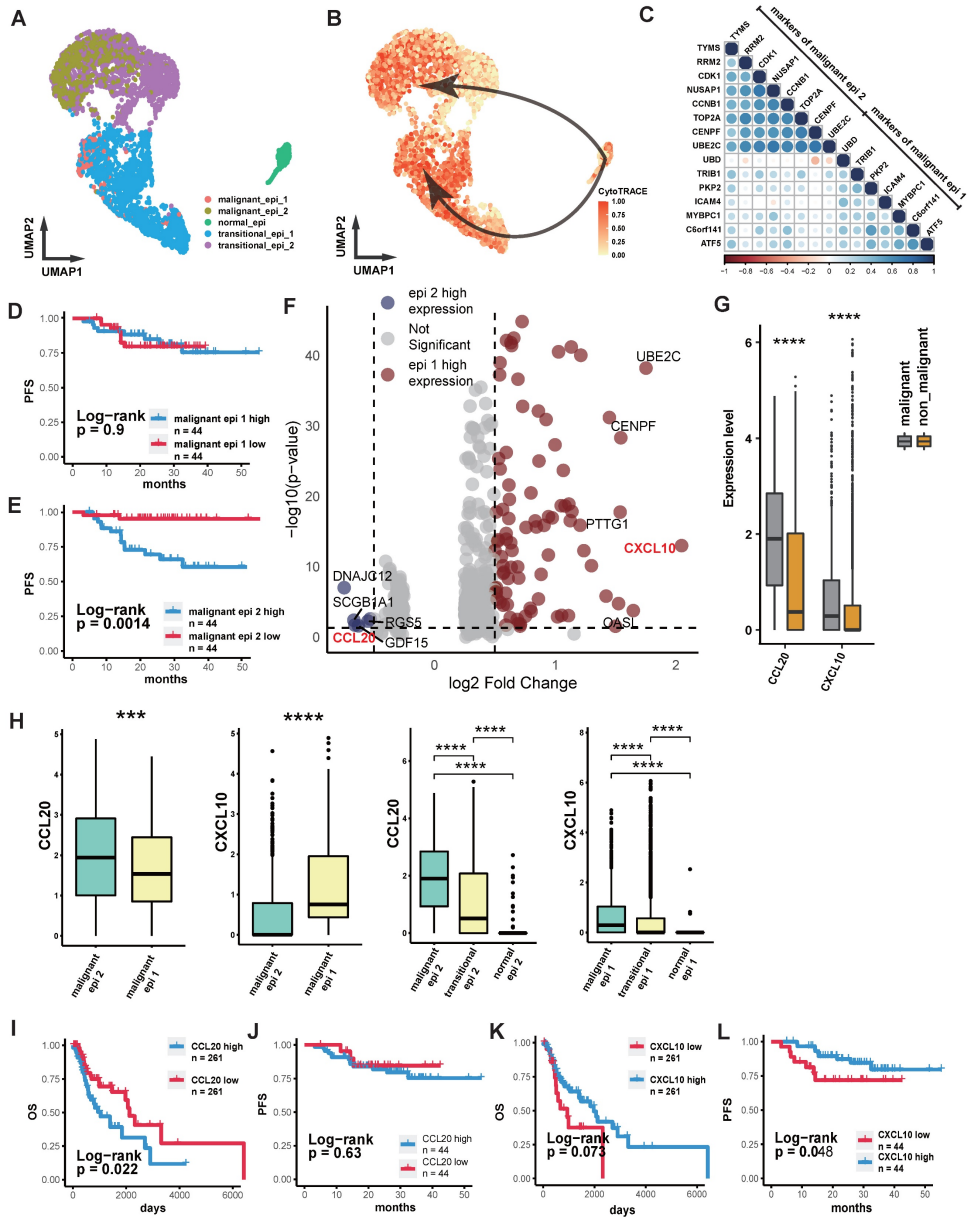
** Intra-tumoral Heterogeneous Subgroups and Chemokine Expression Profiles in NPC. (A)** Scatter plot showing the distribution of epithelial cells after UMAP dimensionality reduction, with different colors representing different clusters. **(B)** The depth of color represents the developmental trajectory inferred by Cytotrace, with darker colors indicating cells closer to the developmental endpoint. Arrows show the cell developmental direction inferred by Slingshot.** (C)** Correlation heatmap showing the correlation of each gene with others in the bulk RNA-seq dataset. Color intensity and bubble size indicate the level of correlation. **(D)-(E)** Kaplan-Meier (K-M) curves illustrating the relationship between the abundance of different malignant epithelial subgroups and progression-free survival (PFS). P-values were derived from log-rank tests.** (F)** The volcano plot illustrates genes that are highly expressed in malignant epithelium 1 (blue) and malignant epithelium 2 (red). **(G)** Box plot comparing the expression levels of chemokines in epithelial cells between malignant and non-malignant epithelium. Significance: ****, P<0.0001; ***, P<0.001; ns, P≥0.05. **(H)** Box plot comparing the expression levels of CXCL10 and CCL20 in two malignant epithelial subgroups or different stages of epithelial evolution. Significance: ****, P<0.0001; ***, P<0.001. **(I)-(J)** K-M curves showing the relationship between high and low expression of CCL20 in HNSCC (H) and OS, and in NPC (I) with PFS. P-values were derived from log-rank tests. **(H)-(L)** K-M curves showing the relationship between high and low expression of CXCL10 in HNSCC (J) and OS, and in NPC (K) with PFS. P-values were derived from log-rank tests.

**Figure 2:**
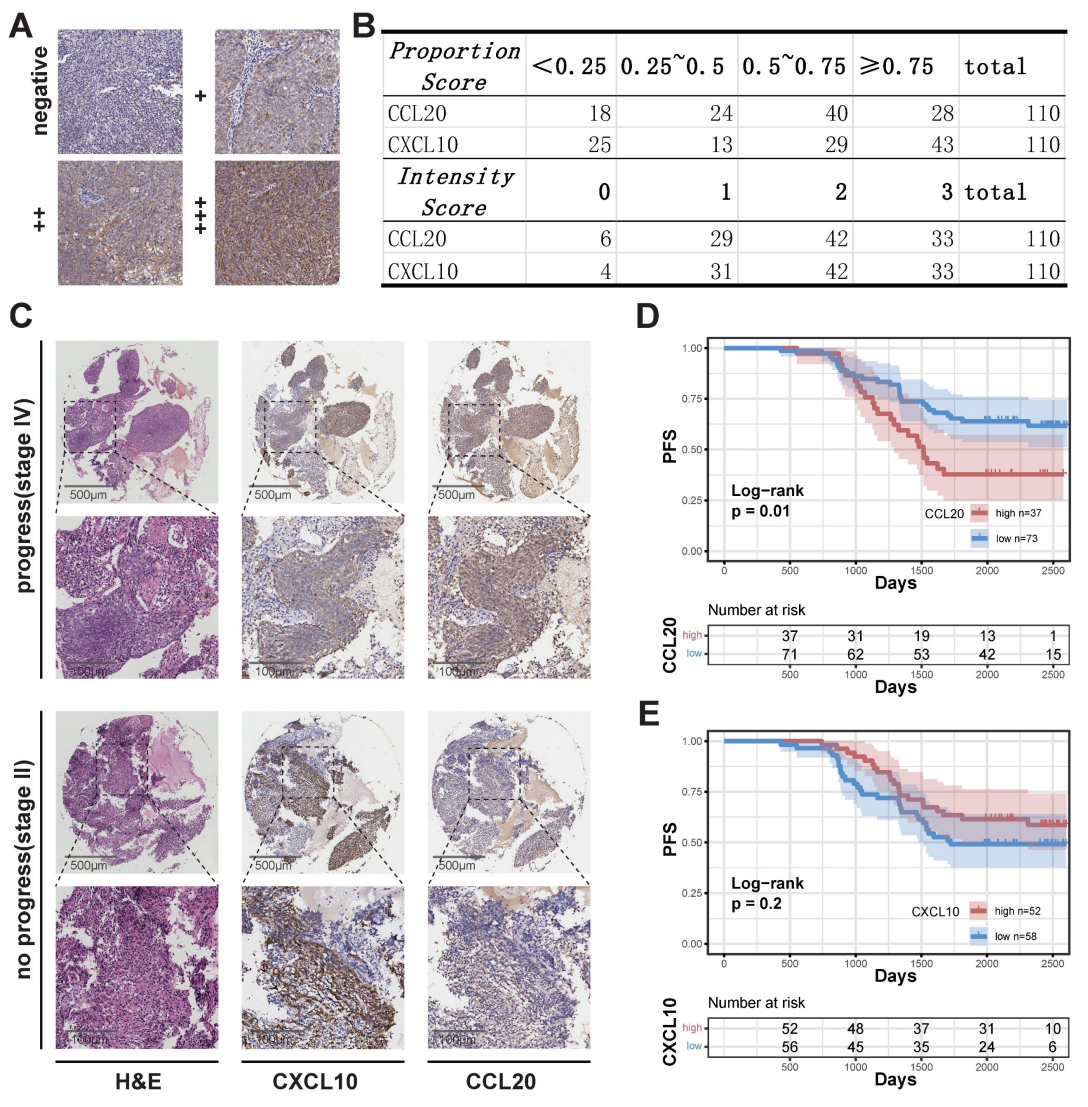
** Association Between CXCL10 and CCL20 Expression and NPC Prognosis. (A)** Staining evaluation criteria. **(B)** Quantitative analysis of grading outcomes. **(C)** Representative H&E staining, CXCL10, and CCL20 immunohistochemical staining of two NPC samples from the progression and non-progression stages.** (D)-(E)** K-M curves showing the relationship between high and low expression of CCL20 (D) and CXCL10 (E) with PFS in NPC. P-values were derived from log-rank tests.

We performed quality control, clustering, annotation, and other analyses on the single-cell data based on our previous study^26^. ScRNA sequencing analysis of epithelial cells showed that normal epithelial cells from nasopharyngeal lymphatic hyperplasia (NLH) clustered independently (Figure 1A). Epithelial cells from NPC clustered into two distinct groups (Figure 1A). These were further categorized into transitional epithelium (translational epi 1 and 2) and malignant epithelium (malignant epi 1 and 2) based on copykat analysis of diploid and aneuploid cells. Developmental trajectory analysis revealed changes from normal epithelium to transitional epithelium and finally to malignant epithelium, with two differentiation pathways (Figure 1B). Characteristic genes from both malignant epithelial clusters were extracted and a correlation matrix was constructed using bulk RNA-seq data, showing independent clustering for each of the two groups (Figure 1C). The characteristic gene score of malignant epi 1 did not show a significant correlation with prognosis (Figure 1D), whereas high characteristic gene scores in malignant epi 1 were associated with significantly poorer prognosis in NPC patients (Figure 1E). This suggests that the two clusters may represent different functional subgroups with heterogeneity. We performed differential gene expression analysis between the two subclusters, which revealed that CXCL10 is highly expressed in malignant epithelium 1, whereas CCL2 is highly expressed in malignant epithelium 2 (Figure 1F; Supplementary data 1). Furthermore, the expression levels of CXCL10 and CCL2 in malignant epithelial cells were significantly higher than those in non-malignant epithelial cells (Figure 1G). CCL20 was specifically highly expressed in malignant epi 2, while CXCL10 was specifically high in malignant epi 1, with both chemokines gradually increasing in expression from normal to transitional and malignant epithelium (Figure 1H). High expression of CCL20 in HNSCC was associated with poorer overall survival (OS) (Figure 1I), and in NPC, it was associated with a trend toward reduced progression-free survival (PFS), though the result did not reach statistical significance (Figure 1J). CXCL10 high expression showed a trend toward prognostic relevance in HNSCC and was indicative of a survival difference in NPC (Figure 1K-L). We subsequently performed IHC staining on NPC tissues to evaluate CCL20 and CXCL10 expression patterns. Both staining *Intensity Scores* and positive cell *Proportion Scores* were systematically assessed (Figure 2A-B). Following our established methodology, we calculated comprehensive *Overall Scores* to stratify NPC patients into high- and low-expression subgroups for subsequent survival analysis (Figure 2D-E). In NPC, progression-stage patients showed elevated tissue expression of CCL20 (Figure 2C), and high CCL20 expression was linked to shorter PFS (Figure 2D), while high CXCL10 expression showed a trend toward association with longer PFS (Figure 2E), though this did not reach statistical significance.

### Differential Expression of CCL20 and CXCL10 in NPC Tumor Cells Leads to Different Anti-tumor Immune States

**Figure 3:**
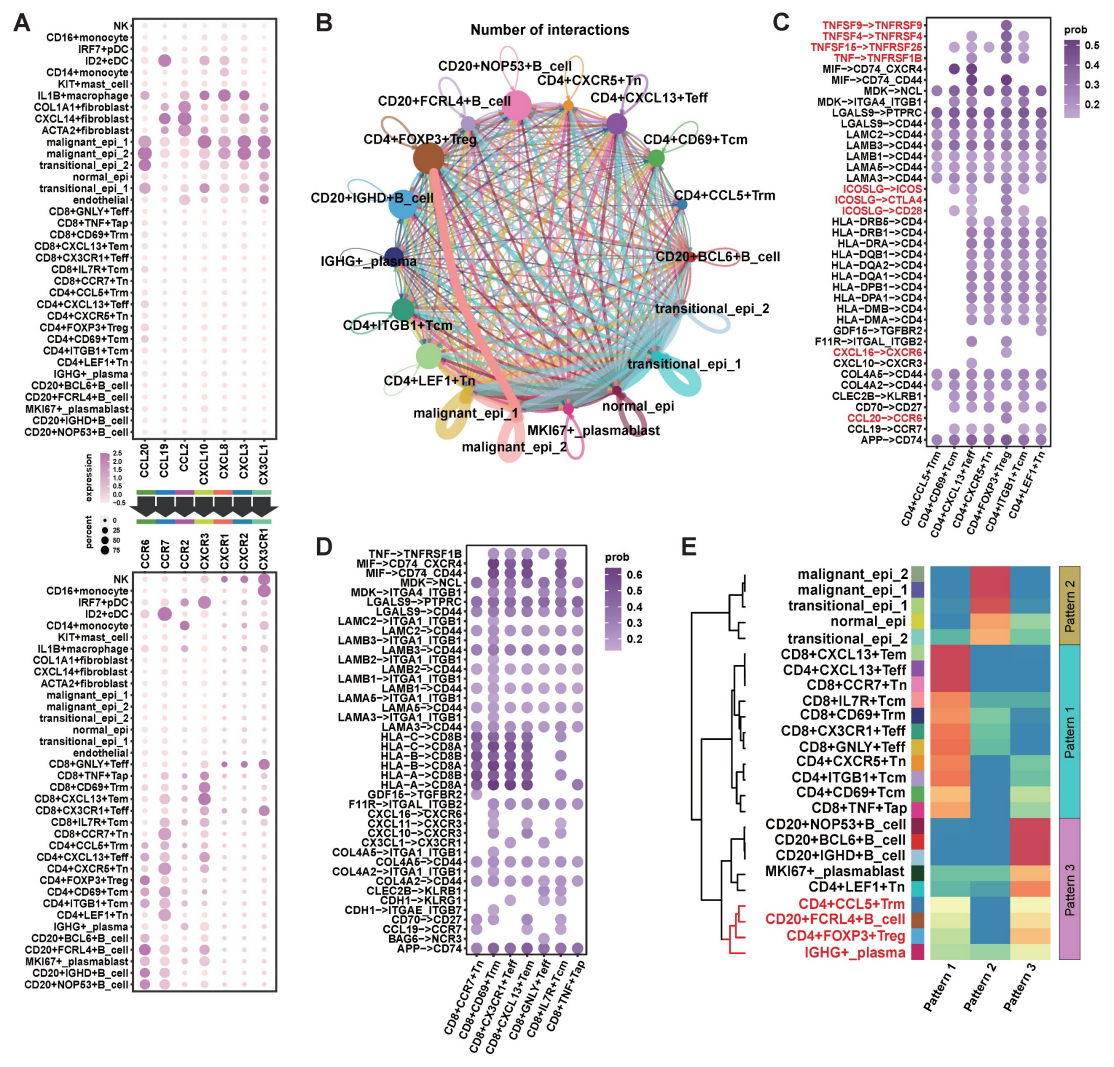
** Communication Between Malignant Epithelium and the TIME in NPC via CXCL10 and CCL20. (A)** Upper half of the bubble plot showing the expression levels of chemokines positively expressed in epithelial cells, and the lower half showing the corresponding ligand expression levels. The size of the bubbles represents the proportion of expression, and the color represents the level of expression. **(B)** CellChat inferred the number of interactions between epithelial cells, CD4+ T cells, and B cells. The thicker the connecting lines between cell subgroups, the more interactions. **(C)** CellChat inferred receptor-ligand pairs for interactions between malignant epithelial 2 and CD4+ T cell subgroups, with color intensity representing the relative strength of interactions. **(D)** CellChat inferred receptor-ligand pairs for interactions between malignant epithelial 1 and CD8+ T cell subgroups, with color intensity representing the relative strength of interactions. **(E)** CellChat inferred signaling reception patterns for epithelial, T cell, and B cell subgroups, identifying three distinct patterns. The heatmap shows the scores for these three patterns, with red indicating high scores and blue indicating low scores. Hierarchical clustering was performed.

Cell communication analysis was performed based on the upregulated chemokines in the malignant epithelium (Figure 1F) and their ligands. The results showed that CCL20 had stronger signaling in malignant epi 2, with its ligands primarily expressed in Treg cells and various B cells (Figure 3A). CXCL10 showed stronger signaling in malignant epi 1, and its ligands were primarily expressed on CD8+ T cells, effector CD4+ T cells, and B cells (Figure 3A). This suggests that malignant epi 2 predominantly recruits Treg cells and various B cell subtypes, while malignant epi 1 mainly attracts CD8+ T cells, effector CD4+ T cells, and B cells.

Additionally, we observed that malignant epi 2 had more interactions with Treg cells compared to malignant epi 1 (Figure 3B). We further analyzed the signals sent by malignant epi 2 to CD4+ T cells (Figure 3C), where tumor necrosis factor (TNF) and its superfamily members (TNFSF) receptor-ligand interactions were significantly elevated in Treg cells. Also, the ICOS signal from malignant epi 2 was stronger in Treg cells (Figure 3C). Regarding chemokines, besides CCL20-CCR6, CXCL16-CXCR6 was also found in the signaling from malignant epi 2 to Treg cells (Figure 3C). CXCR3, expressed at the highest level in CD8+ T cells, showed the strongest potential for being attracted by CXCL10, which was highly expressed in malignant epi 1 (Figure 3A). We then analyzed the signals sent by malignant epi 1 to CD8+ T cells (Figure 3D), with the most notable signals being cell adhesion and antigen presentation. To validate the results obtained from single-cell sequencing, we performed flow cytometry to detect the expression of CCR6 and CXCR3 in immune cells (Supplementary Figure 1). The results showed that CCR6 was primarily expressed in FOXP3+ Tregs and CD20+ B cells (Supplementary Figure 1A, B). On the other hand, CXCR3 was predominantly expressed on the surface of CD8+ T cells and CD20+ B cells (Supplementary Figure 1C, D). These findings are consistent with the sequencing analysis results.

**Figure 4:**
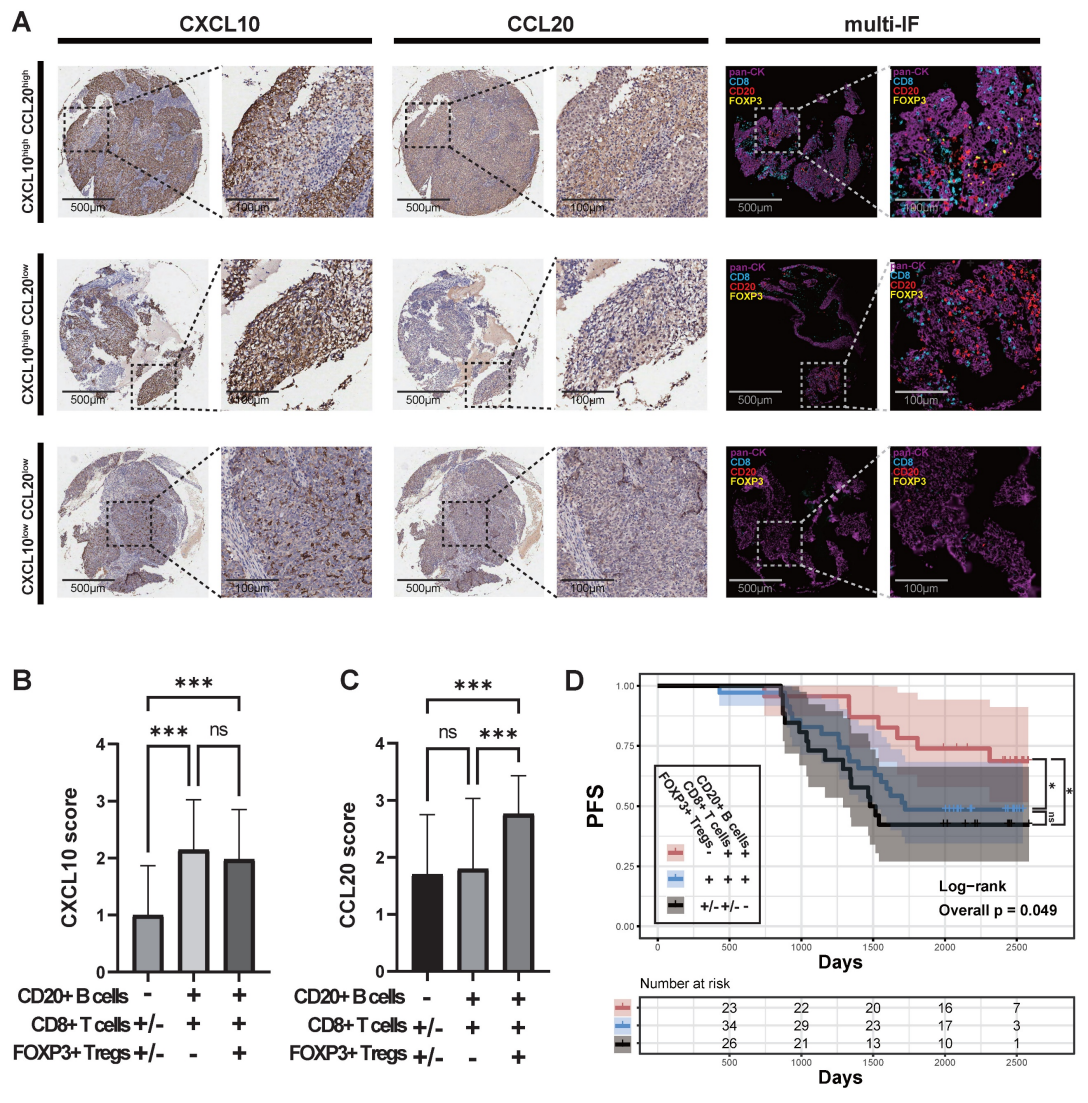
** Immune Cell Infiltration and Its Relationship with CXCL10 and CCL20 in NPC. (A)** Expression of pan-CK, CD8, CD20, and FOXP3 in samples with different levels of CXCL10 and CCL20 expression.** (B)-(C)** CXCL10 and CCL20 scores in samples with different expression states of CD8, CD20, and FOXP3. Significance: ***, P<0.001; ns, P≥0.05. **(D)** PFS of samples with different expression states of CD8, CD20, and FOXP3, with P-values derived from log-rank tests. Significance: *, P<0.05; ns, P≥0.05.

Finally, we clustered the communication patterns between different cell types based on the characteristics of their signal reception (Figure 3E). The results showed that various B cells and Treg cells shared similar signal reception patterns, particularly CD20+FCRL4+B cells, CD4+FOXP3+Treg cells, CD4+CCL5+Trm cells, and plasma cells, which responded to similar signals. In a cohort of 109 primary NPC samples, we observed that when tumor cells highly expressed CCL20, there was a significant infiltration of Treg cells (Figure 4A; Supplementary data 2; Supplementary data 3). When tumor cells highly expressed CXCL10 but lowly expressed CCL20, there was a significant infiltration of CD8+ T cells and CD20+B cells, but no Treg cell infiltration. In patients where both CXCL10 and CCL20 were lowly expressed, an immune desert state was observed. As no samples showed isolated CD20+B cell infiltration, we classified the 109 samples into three categories: 1) lack of CD20+B cell infiltration (CD20-CD8+/-FOXP3+/-); 2) co-infiltration of CD20+B cells and CD8+ T cells (CD20+CD8+FOXP3-); 3) co-infiltration of CD20+B cells, CD8+ T cells, and Treg cells (CD20+CD8+FOXP3+). We found that the CXCL10 score was significantly lower in the lack of CD20+B cell infiltration group (Figure 4B), and the CCL20 score was significantly higher in the CD20+B cell, CD8+ T cell, and Treg cell co-infiltration group (Figure 4C). Survival analysis showed that the lack of CD20+B cell infiltration group had the worst prognosis, the CD20+B cell and CD8+ T cell co-infiltration group had the best prognosis, and the group with Treg cell infiltration in addition to CD20+B and CD8+ T cell co-infiltration had a worse prognosis (Figure 4D). However, there was no significant difference in survival between patients with concurrent infiltration of all three cell types and those without B-cell infiltration (Figure 4D). These results are consistent with the effects of CXCL10 and CCL20 expression on patient prognosis (Figure 2B-C; Figure 4D).

### High Expression of CCL20 in NPC May Induce Immune Escape by Chemotaxis of Treg Cells, Inhibiting Antigen Presentation

**Figure 5:**
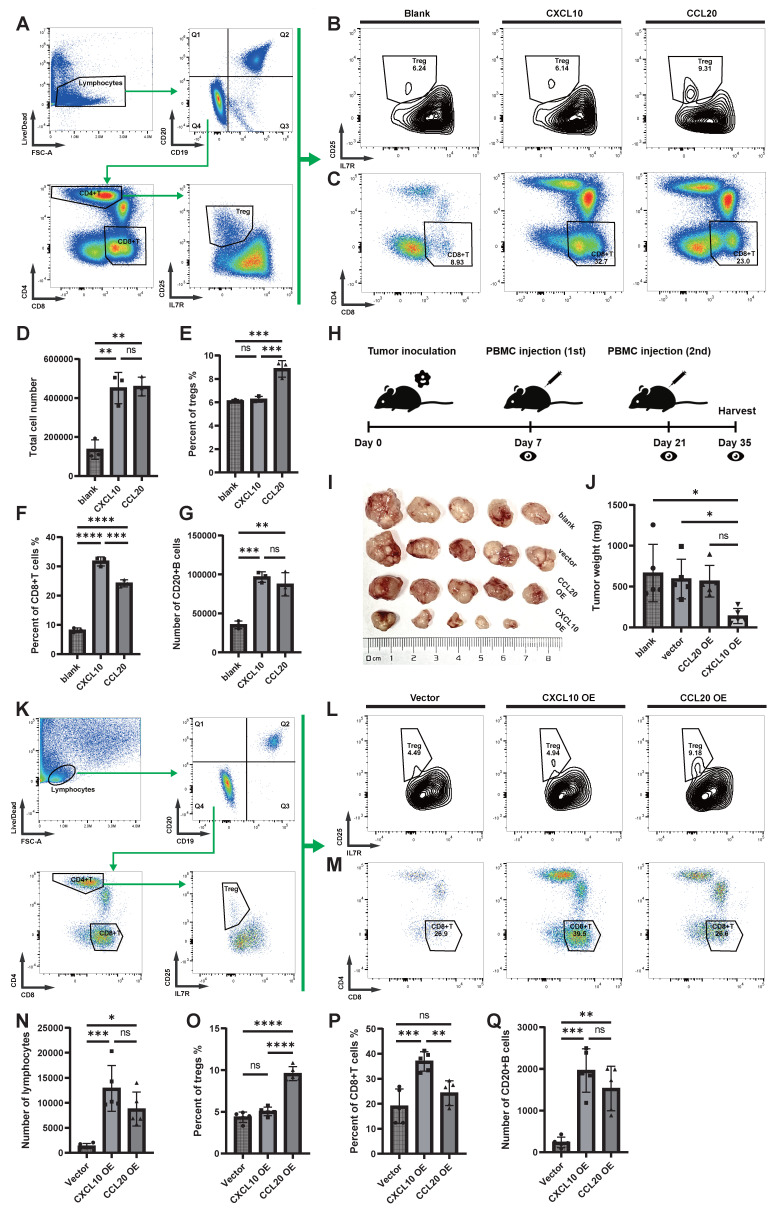
** The Chemotactic Ability of CXCL10 and CCL20 on Immune Cells In Vitro and In Vivo. (A)** Flow cytometry analysis strategy for the in vitro chemotaxis assay. **(B)** Proportion of Treg cells (CD25+IL7R-) within CD4+ T cells. **(C)** Proportion of CD8+ T cells (CD4-CD8+) within T cells (CD19-CD20-). **(D)** Comparison of the total number of migrated cells. Significance: **, P<0.01; ns, P≥0.05. **(E)** Proportion of Treg cells within CD4+ T cells. Significance: ***, P<0.001; ns, P≥0.05. **(F)** Proportion of CD8+ T cells within T cells. Significance: ****, P<0.0001; ***, P<0.001; ns, P≥0.05. **(G)** Comparison of the total number of migrated B cells. Significance: ***, P<0.001; **, P<0.01; ns, P≥0.05. **(H)** Animal experimental protocol: subcutaneous implantation of 5-8F cells, followed by PBMC re-infusion on days 7 and 21, and tumor collection on day 35. **(I)** Tumor growth in different groups. **(J)** Tumor weight in different groups. Significance: *, P<0.05; ns, P≥0.05.** (K)** Flow cytometry analysis strategy after tumor dissection. **(L)** Proportion of Treg cells (CD25+IL7R-) within CD4+ T cells. **(M)** Proportion of CD8+ T cells (CD4-CD8+) within T cells (CD19-CD20-). **(N)** Comparison of the total number of lymphocytes after tumor dissection. Significance: ***, P<0.001; *, P<0.05; ns, P≥0.05.** (O)** Proportion of Treg cells within CD4+ T cells. Significance: ****, P<0.0001; ns, P≥0.05.** (P)** Proportion of CD8+ T cells within T cells. Significance: ***, P<0.001; **, P<0.01; ns, P≥0.05. **(Q)** Comparison of the total number of B cells after tumor dissection. Significance: ***, P<0.001; **, P<0.01; ns, P≥0.05.

The observational studies mentioned above suggest that CCL20 may counteract anti-tumor immunity by recruiting Treg cells. To verify this hypothesis, we designed in vitro chemotaxis experiments and performed flow cytometric analysis on the cell composition after chemotaxis of recombinant CXCL10 and CCL20 on PBMCs (Figure 5A). The total number of cells chemotaxed by CXCL10 and CCL20 was similar but significantly higher than the blank control group (Figure 5D). In the CCL20 group, the proportion of Treg cells among T cells and the number of Treg cells were significantly higher than in the other two groups (Figure 5B, E; Supplementary figure 2A). In the CXCL10 group, the proportion of CD8+ T cells among non-B cells and the number of CD8+T cells were significantly higher than in the other two groups (Figure 5C, F; Supplementary figure 2B). The numbers of CD20+ B cells in both CXCL10 and CCL20 groups were higher than the blank control, but no significant difference was observed between the two groups (Figure 5G; Supplementary figure 2C).

In the animal experiments, we transplanted 5-8F cells, 5-8F cells transfected with an empty vector, and 5-8F cells overexpressing CXCL10 (CXCL10 OE) or CCL20 (CCL20 OE) into the subcutaneous tissue of severe immune-deficient NSG mice and performed immune reconstitution (Figure 5H). The results showed that the tumor growth in the CXCL10 OE group was significantly restricted (Figure 5I-J). Flow cytometric analysis of tumor tissue (Figure 5K) revealed that both the CXCL10 OE and CCL20 OE groups had a large number of immune cell infiltrates, while the empty vector-transfected 5-8F group lacked the ability to induce immune cell infiltration (Figure 5N). The CCL20 OE group induced more Treg cells infiltration (Figure 5L, O; Supplementary figure 2D), while the CXCL10 OE group induced more CD8+ T cells infiltration (Figure 5M, P; Supplementary figure 2E), with no significant difference between the two groups concerning B cell numbers (Figure 5Q; Supplementary figure 2F).

**Figure 6:**
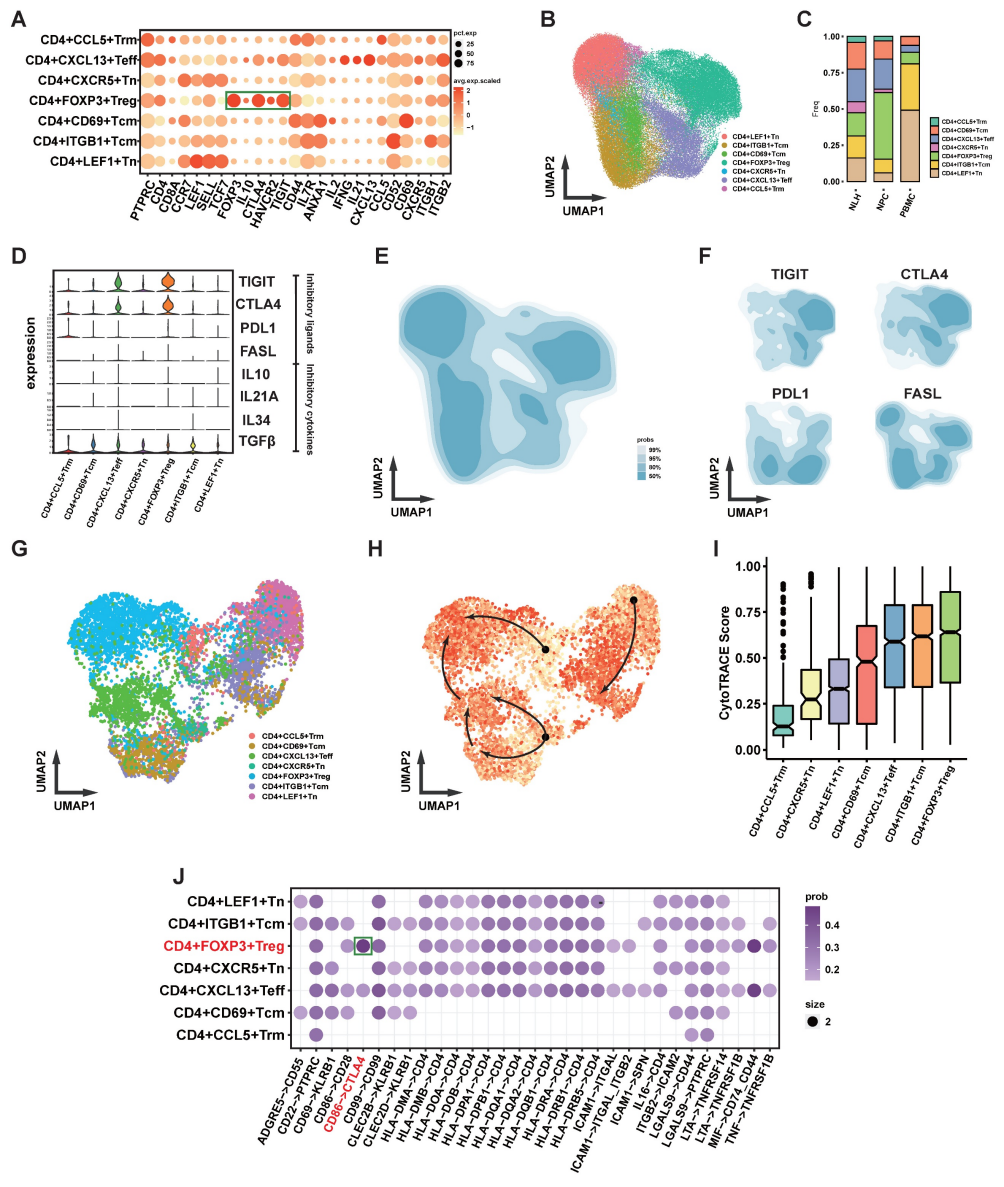
** Functional Analysis of Treg Cells in NPC. (A)** Bubble plot showing the expression levels of functional molecules in different CD4+ T cell subpopulations, with bubble size indicating expression proportion and color representing expression level. **(B)** Scatter plot showing the distribution of CD4+ T cells after UMAP dimensionality reduction, with different colors marking different clusters.** (C)** Stacked bar chart showing the distribution of different CD4+ T cell subpopulations in different samples. **(D)** Violin plot showing the expression levels of inhibitory ligands and cytokines in different CD4+ T cell subpopulations. **(E)-(F)** Density plots showing the distribution density of CD4+ T cells in the UMAP coordinates (E) and the expression density of inhibitory ligands (F). **(G)-(H)** Cytotrace-inferred UMAP coordinates and cell development scores, with darker colors indicating cells closer to the developmental endpoint, and arrows showing the inferred developmental direction.** (I)** Box plot showing the Cytotrace-inferred scores, with higher scores indicating closer proximity to the developmental endpoint. **(J)** Cellchat-inferred receptor-ligand pairs for interactions between B cells and CD4+ T cell subpopulations, with color intensity indicating the relative strength of interactions.

Both in vitro and in vivo experiments suggest that CCL20 has a chemotactic effect on Treg cells, and although the CCL20 OE group recruited more lymphocytes, the tumor growth was not restricted. To explore the potential function of Treg cells, further analysis was performed. In NPC, Treg cells express the classic intracellular marker FOXP3 (Figure 6A), and they are the largest subset of CD4+ T cells (Figure 6B). The proportion of Tregs in NPC is significantly higher compared to PBMC and NLH (Figure 6C). Classic inhibitory cytokines in Tregs were not significantly upregulated, and immunosuppressive ligands such as PD-L1 and FASL were also not notably elevated, while co-inhibitory molecules for antigen presentation, including TIGIT and CTLA4, were significantly upregulated (Figure 6D). We further depicted the distribution density of CD4+ T cells on UMAP coordinates (Figure 6B, E) and the weighted expression density of inhibitory ligands, showing that TIGIT and CTLA4 distribution correlated with that of Tregs (Figure 6F). We also inferred the developmental trajectory of CD4+ T cells (Figure 6G-I), which revealed that Tregs were located at the differentiation endpoint, with their precursors possibly arising from CD4+ CCL5+ Trm cells.

Because Treg and B cells were found to be attracted by similar chemotactic signals in immunohistochemical and cell communication analyses, we conducted a cell communication analysis between CD4+ T cells and B cells (Figure 6J). The results showed that, compared to other CD4+ T cell subsets, the most prominent communication between Tregs and B cells involved the co-inhibitory CTLA4-CD86 signaling.

**Figure 7:**
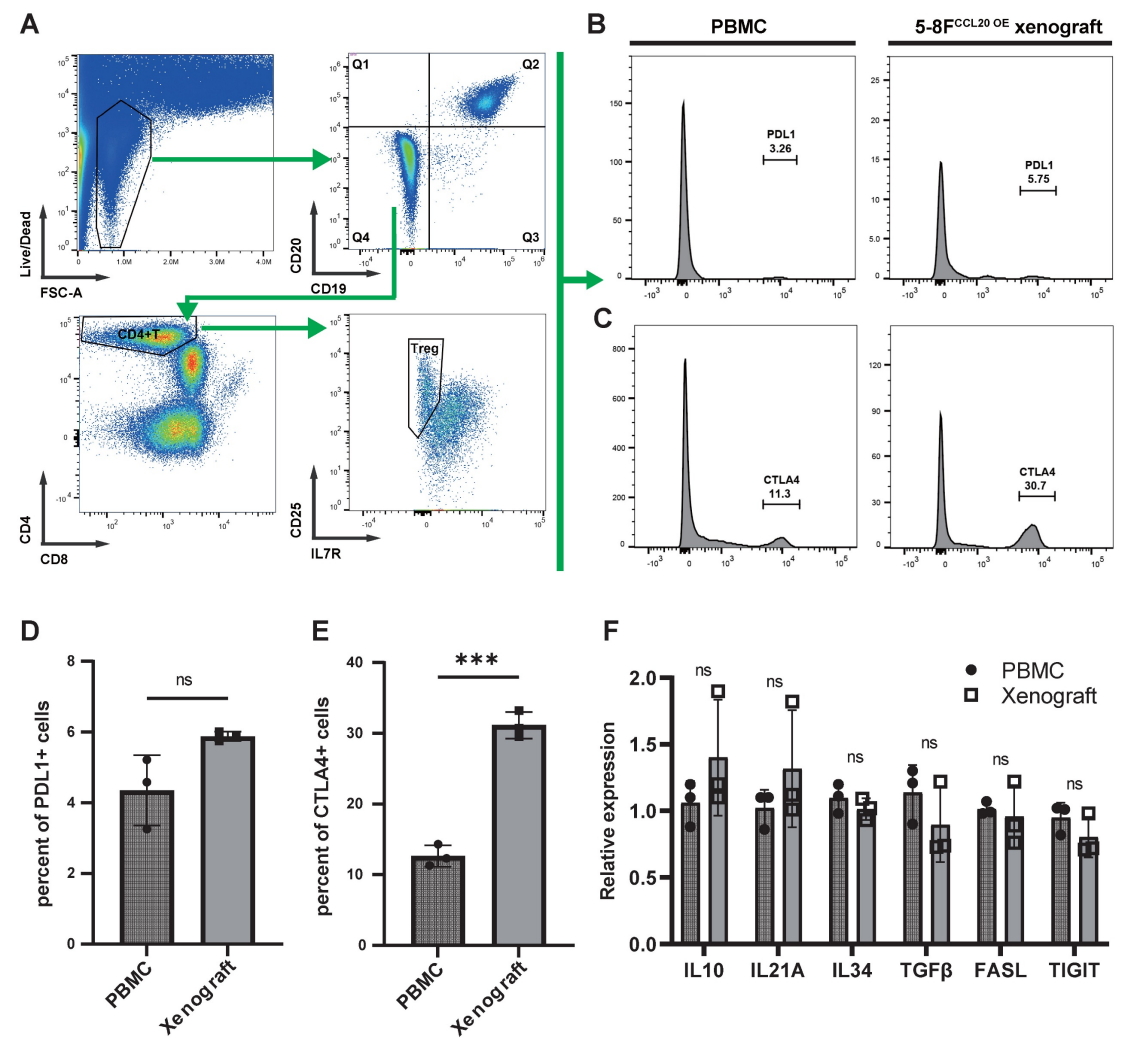
** Expression of Functional Surface Molecules on CCL20-chemotactic Tregs. (A)** Flow cytometry gating strategy. **(B)-(C)** Histograms display the proportions of PD-L1+ (B) and CTLA-4+ (C) cells among Tregs from PBMCs in animal experiments and Tregs chemotactically recruited by CCL20-overexpressing 5-8F cells.** (D)-(E)** Bar graphs compare flow cytometry results. Significance: ***, P<0.001; ns, P>0.05.** (F)** mRNA expression levels of immunosuppressive molecules in Tregs from different samples. Significance: ns, P>0.05.

We next analyzed tumor-infiltrating Tregs in immunoreconstituted mice bearing CCL20-overexpressing 5-8F xenografts, using input human PBMCs from the immunoreconstitution procedure as controls (Figure 7A). Flow cytometry revealed no significant difference in the proportion of PD-L1+ Tregs between tumor-infiltrating Tregs and PBMC-derived Tregs (Figure 7B, D). In contrast, CTLA-4+ Tregs were significantly enriched in tumor-infiltrating Tregs compared to PBMC-derived Tregs (Figure 7C, E). Sorted Tregs were further subjected to PCR analysis, which showed no significant differences in the expression of other immunosuppressive molecules (IL-10, IL-21A, and FASL) between PBMC-derived and tumor-infiltrating Tregs (Figure 7F).

## Discussion

Chemokines play a crucial role in guiding the migration of immune cells, which is essential for establishing an effective anti-tumor immune response. Meanwhile, tumors can also recruit immunosuppressive cells through the secretion of chemokines to facilitate immune evasion^27^, ^28^. As a result, chemokines originating from tumor cells themselves are gaining increasing attention. In this study, high-throughput sequencing was employed to analyze cytokines secreted by NPC tumor cells, revealing CXCL10, which promotes immune responses, and CCL20, which mediates immune suppression. The differential chemotaxis of these chemokines towards immune cells and their impact on survival were validated through both in vivo and in vitro experiments. The implications of their expression in NPC warrant further investigation.

The role of CXCL10 has been extensively elucidated in tumors beyond NPC, with current evidence suggesting that it induces a favorable anti-tumor TIME. CXCL9 and CXCL10 can be produced by antigen-presenting cells, such as dendritic cells or macrophages, as well as by tumor cells^29^. In human non-small cell lung cancer (NSCLC), high CXCL10 expression has been associated with a better response to immune checkpoint inhibitors (ICIs)^30^. In various tumors, the CXCL10/CXCR3 axis has also been found to regulate the migration, differentiation, and activation of immune cells, thereby inhibiting tumor growth^31^. Additionally, CXCL10 is thought to contribute to the development of a "hot" TIME^29^. Furthermore, CXCL10, also known as Interferon gamma-induced protein 10 (IP-10), is strongly induced by IFN-γ and IFN-α/β^31^. This suggests that there may be a positive feedback loop between the release of CXCL10 and effector immune cells, mutually enhancing each other's activity. However, studies on the significance of CXCL10 expression in NPC are limited. In the few available studies, authors have suggested, based on relatively weak evidence, that CXCL10 is associated with poor prognosis^32^, ^33^. This finding contradicts the conclusions of our study, indicating that further investigation may be needed.

Regarding CCL20, similar to our findings in NPC, its high expression has been confirmed to be associated with poorer prognosis in various cancers, including hepatocellular carcinoma (HCC)^34^, breast cancer^35^, colorectal cancer (CRC)^36^, and pancreatic cancer^37^. CCL20 can be secreted by various immune cells, and in prostate^38^ and gastric cancers^39^, it is also abundantly produced by tumor cells. Additionally, a study on CCL20 in CRC found that CCL20 secreted by CRC cells can recruit Treg cells through the FOXO1/CEBPB/NF-κB signaling pathway, thereby promoting chemotherapy resistance^36^. This is consistent with our findings, as we observed that CCL20 could chemotactically attract a higher proportion of Tregs in vitro, and in mouse models, the transplantation of NPC cells overexpressing CCL20 led to an increased infiltration of Tregs. At the same time, although CCL20 induced more lymphocyte infiltration in animal experiments, it did not limit tumor growth. In contrast, CXCL10 induced a similar level of lymphocyte infiltration, but tumor growth was significantly restricted. These findings suggest that the immunosuppressive effects of CCL20 are likely mediated through Treg cells. In one of our previous studies, we found that CD20+FCRL4+ B cells play a crucial role in the anti-tumor immune response in NPC^26^. Treg cells and CD20+FCRL4+ B cells share a similar chemotactic signaling receptor pattern, which allows them to be attracted by CCL20. Moreover, functional analysis of Tregs in NPC revealed that their immunosuppressive activity is primarily mediated by co-inhibitory molecules such as CTLA4 and TIGIT. Therefore, we speculate that Tregs may facilitate tumor immune evasion by masking antigen presentation signals.

The CCL20-CCR6 chemokine axis has not yet been reported to play a role in Treg recruitment in the context of this study. However, it is well established that Treg homing depends on specific chemokine-receptor interactions. Among these, the CCL17/CCL22-CCR4 axis is one of the most clearly defined pathways for Treg chemotaxis. Numerous studies have demonstrated that dendritic cells (DCs) and tumor-associated macrophages (TAMs) are the primary sources of CCL17 and CCL22, and that this axis plays a critical role in Treg recruitment in various solid tumors, including non-small cell lung cancer^40^, breast cancer^41^, and lymphomas^42^. Notably, CCR4 expression is highly enriched in Treg cells, providing a key molecular basis for their targeted migration.

In our study, we observed a similar phenomenon in single-cell transcriptomic data from nasopharyngeal carcinoma (NPC). We systematically analyzed the expression patterns of all chemokines and their receptors across different cell types (see Supplementary Figure 3). As expected, CCR4 was strongly enriched in Treg cells, while CCL17 and CCL22 were predominantly expressed by DCs, suggesting that Treg recruitment in NPC is dependent on the activity of antigen-presenting cells and may reflect a negative feedback loop in immune regulation. In addition to this classical axis, recent studies have highlighted the CCL1/CCL18-CCR8 axis as another important mechanism for Treg recruitment, particularly in epithelial tumors^43^. In our data, CCR8 was also highly expressed in Treg cells. However, intriguingly, CCL1 and CCL18 were barely detectable in NPC tissues, suggesting that this pathway may not play a major role in Treg recruitment in NPC.

Moreover, we observed that among all chemokines analyzed, CCL20 was uniquely and markedly expressed by tumor cells. This finding raises the possibility that CCL20 serves as a tumor-derived chemokine actively involved in recruiting immunosuppressive cells such as Tregs. Unlike other chemokines that are primarily secreted by immune cells, CCL20 expression by tumor cells may represent a distinct mechanism of tumor-intrinsic immune evasion, which warrants further investigation.

In summary, we found that CXCL10 and CCL20 in NPC primarily originate from tumor cells and may represent a pair of antagonistic cytokines. CXCL10 promotes anti-tumor immunity by chemotactically attracting effector immune cells, while CCL20 likely recruits Tregs, inhibiting the function of effector immune cells.

## Supplementary Material

Supplementary figures and data.
