# Supplementary material for: Tumor-Derived CXCL10 and CCL20 Polarize the Immune Microenvironment in Nasopharyngeal Carcinoma via Competitive Recruitment of Effector T Cells and Tregs
Source: Int J Med Sci. 2026 Jan 1;23(1):26–41. doi: 10.7150/ijms.116010 (PMC12701978; doi:10.7150/ijms.116010)
Supplement: Supplementary file 1 — Supplementary figures and data. [file ijmsv23p0026s1.zip › supplementary figures.pdf]

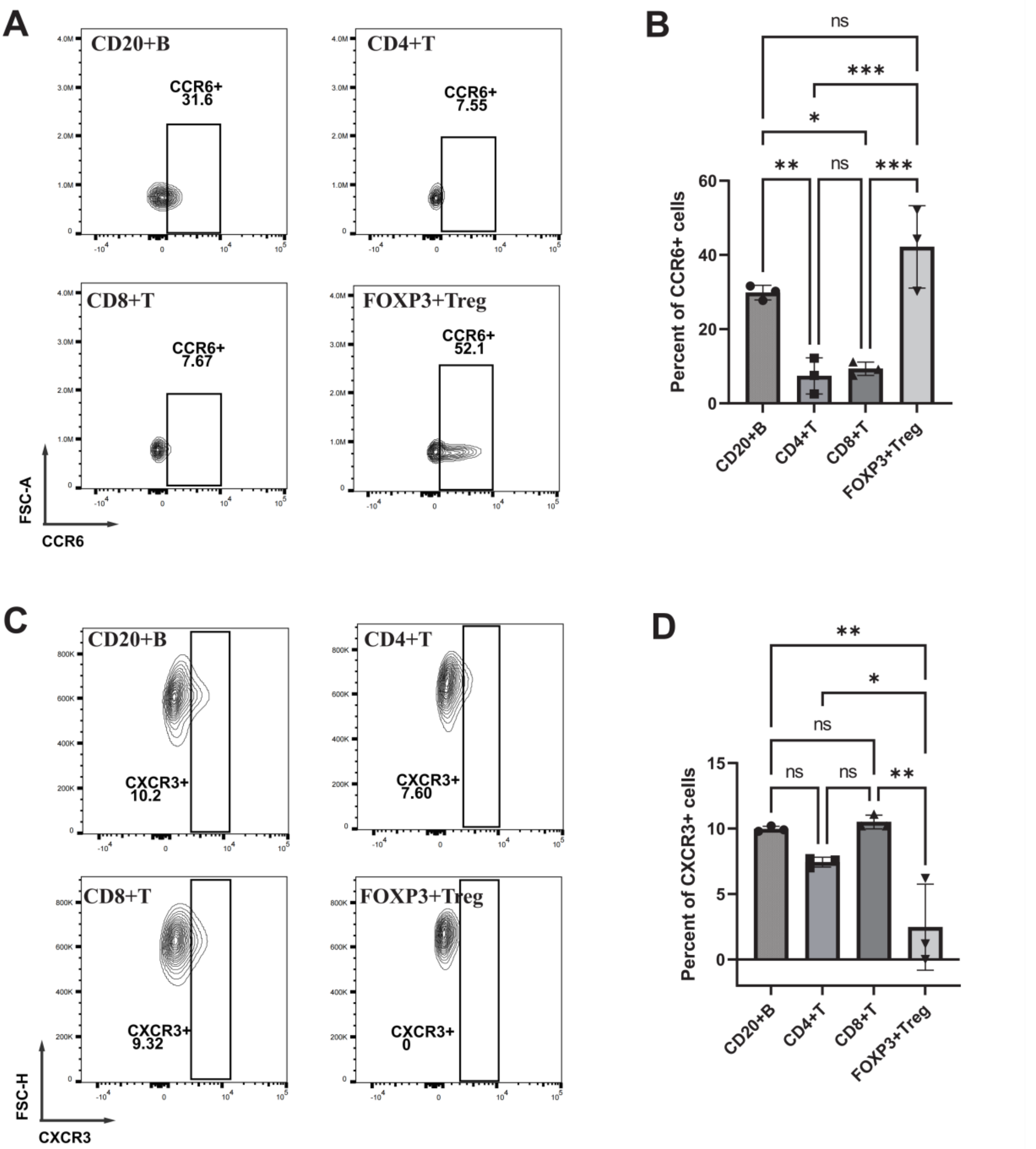

Supplementary Figure 1. Expression of chemokine receptors in distinct lymphocyte populations. (A) Flow cytometric analysis of CCR6 expression levels, with gated populations representing positive cells. **(B)** Bar graph comparing the percentages (%) of CCR6-positive cells among different lymphocyte subsets. Significance: ***, P<0.001; **, P<0.01; *, P<0.05; ns, P>0.05. **(C)** Flow cytometric analysis of CXCR3 expression levels, with gated populations representing positive cells. **(D)** Bar graph comparing the percentages (%) of CXCR3-positive cells among different

lymphocyte subsets. Significance: **, P<0.01; *, P<0.05; ns, P>0.05.

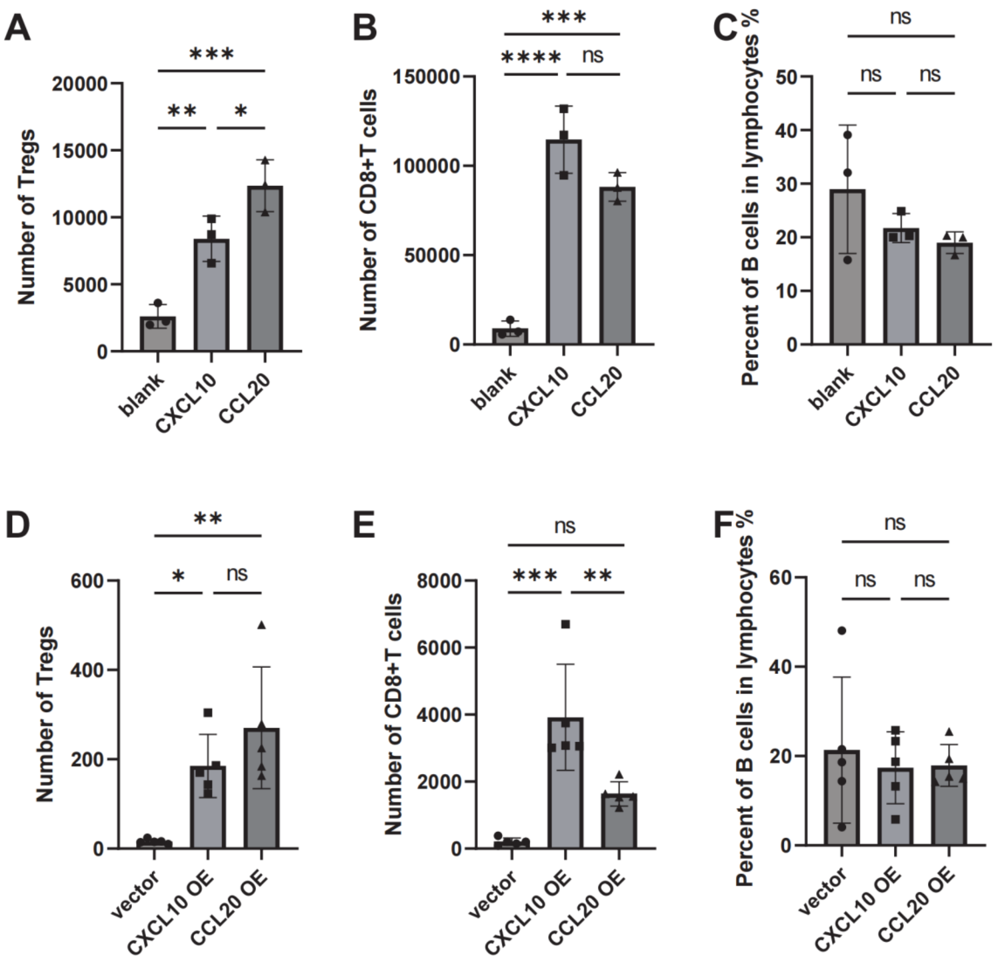

Supplementary figure 2. The Chemotactic Ability of CXCL10 and CCL20 on Immune Cells In Vitro and In Vivo. (A) Number of Treg cells in in vitro chemotactic experiment. Significance: ***, P<0.001; **, P<0.01; *, P<0.05. **(B)** Number of CD8+ T cells in in vitro chemotactic experiment. Significance: ****, P<0.0001; ***, P<0.001; ns, P≥0.05. **(C)** Comparison of the proportion of migrated B cells with lymphocytes in in vitro chemotactic experiment. Significance: ns, P≥0.05. **(D)** Number of Treg cells in in vivo chemotactic experiment. Significance: **, P<0.01; *, P<0.05; ns, P≥0.05. **(E)** Number of CD8+ T cells in in vivo chemotactic experiment. Significance: ***, P<0.001;

, P<0.01; ns, P≥0.05. **(F) Comparison of the proportion of migrated B cells with lymphocytes in in vivo chemotactic experiment. Significance: ns, P≥0.05.

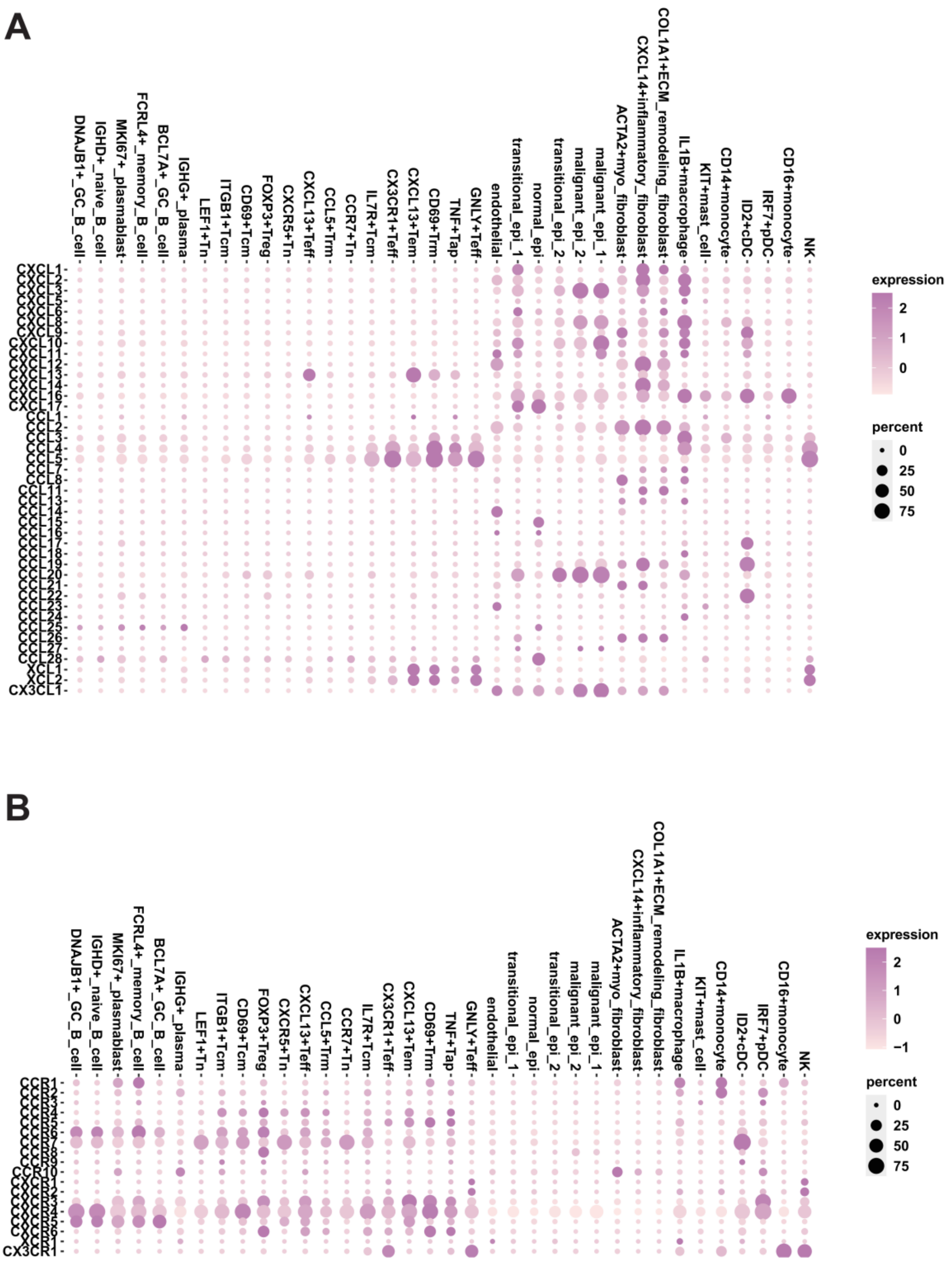

Supplementary figure 3. The expression of chemokines and chemokine receptors in different cell subpopulations. (A) Bubble plot showing the expression levels of

chemokines positively expressed in different cell types, and **(B)** showing the corresponding ligand expression levels. The size of the bubbles represents the proportion of expression, and the color represents the level of expression.
